# Deucravacitinib, an Oral, Selective, Allosteric Tyrosine Kinase 2 Inhibitor, in Japanese Patients With Plaque Psoriasis: In‐Depth Analysis of Efficacy and Safety in the Phase 3 POETYK PSO‐4 Trial

**DOI:** 10.1111/1346-8138.17744

**Published:** 2025-04-30

**Authors:** Yukari Okubo, Akimichi Morita, Shinichi Imafuku, Yayoi Tada, Katsuki Tsuritani, Yanqiu Shao, Zoran Popmihajlov, Andrew Napoli, Lauren Hippeli, Katsuyoshi Habiro, Mamitaro Ohtsuki

**Affiliations:** ^1^ Department of Dermatology Tokyo Medical University Tokyo Japan; ^2^ Department of Geriatric and Environmental Dermatology Nagoya City University Graduate School of Medical Sciences Nagoya Japan; ^3^ Department of Dermatology Faculty of Medicine, Fukuoka University Hospital Fukuoka Japan; ^4^ Department of Dermatology Teikyo University School of Medicine Tokyo Japan; ^5^ Immunology Medical Strategy Bristol Myers Squibb K.K. Tokyo Japan; ^6^ Biometrics Bristol Myers Squibb Princeton New Jersey USA; ^7^ Clinical Development Bristol Myers Squibb Princeton New Jersey USA; ^8^ WW Medical I&F Bristol Myers Squibb Princeton New Jersey USA; ^9^ Department of Dermatology Jichi Medical University Tochigi Japan

**Keywords:** biologic therapy, deucravacitinib, hard‐to‐treat psoriasis, PASI, treat‐to‐target threshold

## Abstract

Deucravacitinib, an oral, selective, allosteric tyrosine kinase 2 inhibitor, is approved in Japan for adults with plaque, generalized pustular, and erythrodermic psoriasis who have inadequate response to conventional systemic therapies. In the Phase 3, open‐label POETYK PSO‐4 (NCT03924427) trial, deucravacitinib was efficacious and well tolerated in Japanese patients with moderate to severe plaque psoriasis. This post hoc analysis of PSO‐4 evaluated deucravacitinib efficacy and safety in greater detail in this patient population. Absolute Psoriasis Area and Severity Index (PASI), achievement of PASI thresholds of ≤ 1, ≤ 2, and ≤ 5, and PASI body region (head, trunk, upper limbs, lower limbs) and plaque characteristic (erythema, induration, desquamation) scores were evaluated over 52 weeks. Response rates (PASI 75, PASI 90, and static Physician Global Assessment score of 0 [clear] or 1 [almost clear]) were evaluated based on prior use of systemic (biologic and nonbiologic) therapy and phototherapy. Efficacy was also evaluated in patients with scalp, fingernail, and palmoplantar psoriasis. Select safety events were reviewed. Deucravacitinib improved absolute PASI from Week 1, with improvements maintained through Week 52. Deucravacitinib‐treated patients achieved clinically meaningful improvements in PASI thresholds, with nearly half (47.6%) achieving PASI ≤ 1 at Week 52. Deucravacitinib also improved PASI body region and plaque characteristic scores, with improvements that occurred as early as Week 1 maintained through Week 52. Deucravacitinib was efficacious through Week 52 regardless of prior use of systemic therapy or phototherapy. Deucravacitinib was also efficacious in patients with scalp and fingernail psoriasis, and in the limited number with palmoplantar psoriasis. Serious adverse events, adverse events resulting in discontinuation, and shifts to Grade ≥ 3 laboratory abnormalities were rare over 52 weeks. This analysis provides a more detailed characterization of Japanese patients with plaque psoriasis appropriate for deucravacitinib treatment and confirms that deucravacitinib is efficacious and well tolerated in this patient population.

**Trial Registration:** www.ClinicalTrials.gov identifier: NCT03924427

## 1 Introduction

Psoriasis is a chronic, immune‐mediated, inflammatory disease that reduces health‐related quality of life [1]. The prevalence rate of psoriasis in Japan is 0.3%, with plaque psoriasis accounting for 97.4% of cases [2]. Tyrosine kinase 2 (TYK2) is an intracellular mediator of cytokine signaling (e.g., interleukin‐23, interleukin‐12, Type I interferons); psoriasis pathogenesis involves interleukin‐23 and Type 1 interferon signaling [3, 4]. Deucravacitinib, an oral, selective, allosteric TYK2 inhibitor, is approved in the United States, European Union, Japan, South Korea, China, and other countries for the treatment of adults with moderate to severe plaque psoriasis who are candidates for systemic therapy [5, 6, 7, 8, 9, 10, 11]. In contrast to Janus kinase (JAK) 1,2,3 inhibitors that bind to the more conserved catalytic domains of their respective kinases, deucravacitinib uniquely binds to the less conserved regulatory (pseudokinase) domain of TYK2 [3, 12], driving its selectivity for TYK2 and representing the first in a new class of oral drugs.

In POETYK PSO‐1 (NCT03624127) and POETYK PSO‐2 (NCT03611751), two global, Phase 3, double‐blinded, randomized trials, deucravacitinib was superior to placebo and apremilast based on the coprimary endpoints at Week 16 of ≥ 75% reduction from baseline in the Psoriasis Area and Severity Index (PASI 75) and a static Physician Global Assessment score of 0 (clear) or 1 (almost clear) with a ≥ 2‐point improvement from baseline (sPGA 0/1), and was well tolerated in patients with moderate to severe plaque psoriasis [13, 14]. In the Phase 3, open‐label POETYK PSO‐4 (NCT03924427) trial, deucravacitinib was efficacious and well tolerated over 52 weeks in Japanese patients with moderate to severe plaque psoriasis and in the limited number of patients with generalized pustular psoriasis or erythrodermic psoriasis enrolled in this trial [15].

Primary endpoint outcomes based on percent reductions from baseline in PASI (e.g., PASI 75) have frequently been used in clinical trials to evaluate the efficacy of psoriasis therapies [16]. However, patients with moderate to severe disease may achieve relative percent reductions in PASI but continue to experience substantial disease activity and reductions in health‐related quality of life [16]. In contrast, achievement of sustained low absolute PASI may be more clinically meaningful to patients and healthcare providers than a defined relative percent reduction in PASI assessed at a specific time point [16]. Achievement of an absolute PASI threshold ≤ 2 or ≤ 3 is recommended by multiple international dermatology expert consensus panels for patients with moderate to severe plaque psoriasis; an absolute PASI threshold ≤ 2 is considered the treatment goal in the Japanese guidance [17, 18, 19, 20, 21]. Absolute PASI ≤ 2 corresponds to low clinical disease activity and improved health‐related quality of life, and is considered a relevant treat‐to‐target threshold for patients with plaque psoriasis in a real‐world setting [16, 22]. Treat‐to‐target threshold strategies have been shown to be feasible, clinically meaningful, and cost effective in various immune‐mediated diseases, including psoriatic arthritis, rheumatoid arthritis, and inflammatory bowel disease [23]. Psoriatic lesions typically appear on the trunk, scalp, and upper and lower limbs, with lesions on palms and fingernails occurring less frequently [24]. However, patients with psoriatic lesions on the head or neck experience larger reductions in health‐related quality of life because of the greater visibility of their lesions [24].

Although overall efficacy and safety of deucravacitinib have been established in several Phase 3 trials in patients with moderate to severe plaque psoriasis [13, 14, 15], a more detailed characterization of patient populations appropriate for deucravacitinib treatment is needed. The aim of this post hoc analysis of POETYK PSO‐4 was to further clarify appropriate patient profiles by conducting detailed efficacy and safety analyses in Japanese patients with various baseline characteristics.

## 2 Methods

### 2.1 Study Design

The design of the POETYK PSO‐4 trial has been described previously [15]. Briefly, adults (≥ 20 years of age) with moderate to severe plaque psoriasis (PASI ≥ 12, sPGA ≥ 3, and body surface area [BSA] involvement ≥ 10% at baseline) received open‐label treatment with deucravacitinib 6 mg once daily for 52 weeks. POETYK PSO‐4 also enrolled patients with generalized pustular psoriasis and erythrodermic psoriasis; however, these patients were not included in this analysis due to the limited numbers in each population.

### 2.2 Assessments

The PASI measures the severity and extent of psoriasis. First, each body region (head, trunk, upper limbs, and lower limbs) receives a grade for psoriasis intensity (erythema, induration, and desquamation) ranging from 0 (none) to 4 (very severe) [25, 26, 27]. Next, the BSA affected by psoriasis in each region is measured, yielding a grade from 0 (no involvement) to 6 (≥ 90% involvement). For each body region, the psoriasis intensity scores are added to calculate a subtotal; this subtotal is multiplied by the BSA score for that region, the result is multiplied by the weight of the region (0.1 for head and neck, 0.2 for upper limbs, 0.3 for trunk, and 0.4 for lower limbs), and the scores from all four body regions are combined to calculate the total score [25, 26, 27]. The total PASI score ranges from 0 to 72, with higher scores denoting more severe disease activity [25, 26, 27].

Efficacy assessments described in this publication included mean percent change from baseline in absolute PASI over 52 weeks; achievement of absolute PASI thresholds of ≤ 1, ≤ 2, and ≤ 5 at Weeks 16, 24, and 52; mean percent change from baseline in the individual PASI components of body region (head, trunk, upper limbs, and lower limbs) and plaque characteristic (erythema, induration, and desquamation) over 52 weeks; and ≥ 75% reduction from baseline in each PASI body region and plaque characteristic score at Weeks 16, 24, and 52. Additional efficacy assessments included PASI 75, ≥ 90% reduction from baseline in PASI (PASI 90), and sPGA 0/1 by prior therapy (systemic [biologic and nonbiologic] therapy and phototherapy) over 52 weeks as well as outcomes in scalp psoriasis (scalp‐specific Physician Global Assessment score of 0 or 1 [ss‐PGA 0/1]; mean change from baseline and mean percent change from baseline in the Psoriasis Scalp Severity Index [PSSI]), fingernail psoriasis (Physician Global Assessment‐Fingernails score of 0 or 1 [PGA‐F 0/1]; mean change from baseline and mean percent change from baseline in the modified Nail Psoriasis Severity Index [mNAPSI]), and palmoplantar psoriasis (palmoplantar Physician Global Assessment score of 0 or 1 [pp‐PGA 0/1]; mean change from baseline and mean percent change from baseline in palmoplantar PASI [pp‐PASI]) over 52 weeks.

Safety assessments also were evaluated over 52 weeks. These assessments included serious adverse events (SAEs), adverse events (AEs) resulting in treatment discontinuation, and shifts from baseline to Common Terminology Criteria for Adverse Events (CTCAE) Grade ≥ 3 laboratory abnormalities.

### 2.3 Statistical Analysis

Efficacy and safety analyses described in this publication were conducted using the as‐treated population (all enrolled patients who received at least one dose of deucravacitinib treatment). Nonresponder imputation (NRI) was used to account for missing data for binary outcomes in patients who discontinued treatment prior to Week 52 or had missing Week 52 data. Modified baseline observation carried forward (mBOCF) was used to impute missing data for continuous outcomes. Scalp, fingernail, and palmoplantar outcomes were as observed (i.e., no data imputation was performed). As this was a single‐arm trial, statistical testing of treatment comparisons was not performed. SAEs and treatment discontinuations due to AEs are presented in narrative form, and CTCAE Grade ≥ 3 laboratory abnormalities are presented as frequencies and percentages.

## 3 Results

### 3.1 Patient Disposition and Characteristics

In total, 63 patients with plaque psoriasis received deucravacitinib treatment (as‐treated population) and 59 patients completed 52 weeks of treatment. Reasons for not completing treatment were AEs (*n* = 2), lack of efficacy (*n* = 1), and patient withdrawal (*n* = 1). As previously reported, baseline patient demographics and clinical characteristics were typical of patients with moderate to severe plaque psoriasis; 76.2% of patients were male, the mean age of the total population was 49.1 years, and the mean duration of disease in the total population was 15.4 years (Table 1) [15]. The mean baseline PASI was 21.1, the mean baseline BSA involvement was 30.3%, and 88.9% of patients had a baseline sPGA score of 3 (moderate disease) and 11.1% had a baseline sPGA score of 4 (severe disease) [15]. The majority of patients had previously received systemic therapy for plaque psoriasis (biologic, 15.9%; nonbiologic, 55.6%) [15].

**TABLE 1:** Baseline patient demographics and disease characteristics [15].

Parameter	Plaque psoriasis (*n* = 63)
Age, mean (SD), year	49.1 (12.1)
Weight, mean (SD), kg	69.5 (14.2)
BMI, mean (SD), kg/m^2^	24.9 (4.4)
Sex	
Female, *n* (%)	15 (23.8)
Male, *n* (%)	48 (76.2)
Disease duration, mean (SD), year	15.4 (10.7)
Prior systemic treatment, *n* (%)	
Biologic	10 (15.9)
Nonbiologic	35 (55.6)
No prior systemic therapy	18 (28.6)
Phototherapy, *n* (%)	27 (42.9)
PASI, mean (SD)	21.1 (9.2)
sPGA, *n* (%)	
0 (clear)	0
1 (almost clear)	0
2 (mild)	0
3 (moderate)	56 (88.9)
4 (severe)	7 (11.1)
BSA involvement, mean (SD), %	30.3 (18.6)
PSSD symptom score, mean (SD)	39.8 (23.6)
DLQI, mean (SD)	9.1 (4.5)
ss‐PGA ≥ 3, *n* (%)	35 (55.6)
PSSI score, mean (SD)^a^	32.4 (14.9)
PGA‐F ≥ 3, *n* (%)	10 (15.9)
mNAPSI score, mean (SD)^b^	29.7 (12.3)
pp‐PGA ≥ 3, *n* (%)	4 (6.3)
pp‐PASI, mean (SD)^c^	24.0 (22.2)

Abbreviations: BMI, body mass index; BSA, body surface area; DLQI, Dermatology Life Quality Index; mNAPSI, modified Nail Psoriasis Severity Index; PASI, Psoriasis Area and Severity Index; PGA‐F, Physician's Global Assessment–Fingernail; pp‐PASI, palmoplantar Psoriasis Area and Severity Index; pp‐PGA, palmoplantar Physician's Global Assessment; PSSD, Psoriasis Symptoms and Signs Diary; PSSI, Psoriasis Scalp Severity Index; SD, standard deviation; sPGA, static Physician's Global Assessment; ss‐PGA, scalp‐specific Physician's Global Assessment.

^a^ In patients with baseline ss‐PGA score ≥ 3.

^b^ In patients with baseline PGA‐F score ≥ 3.

^c^ In patients with baseline pp‐PGA score ≥ 3.

### 3.2 Efficacy

#### 3.2.1 Effect of Deucravacitinib Treatment on Absolute PASI

**FIGURE 1:**
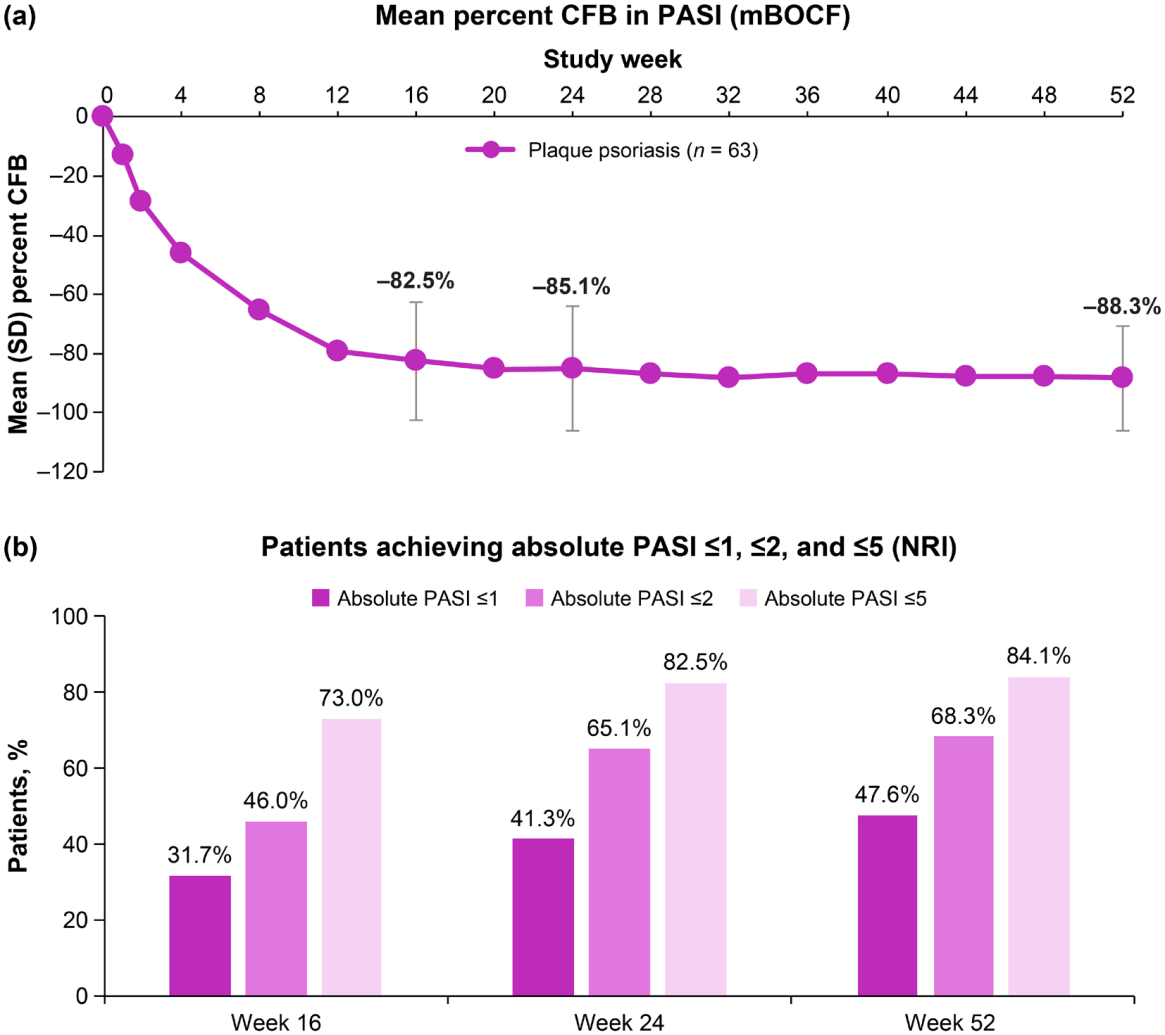
(a) Mean percent change from baseline in absolute PASI (*n* = 63). (b) Proportion of patients achieving absolute PASI treat‐to‐target thresholds (*n* = 63). Nonresponder imputation and modified baseline observation carried forward were used to impute missing data. CFB, change from baseline; mBOCF, modified baseline observation carried forward; NRI, nonresponder imputation; PASI, Psoriasis Area and Severity Index; SD, standard deviation.

The mean percent change from baseline in absolute PASI decreased from Week 1 through Week 16, and further reductions occurred through Week 52 (Figure 1A). Percentages of patients achieving PASI treat‐to‐target thresholds of ≤ 1, ≤ 2, and ≤ 5 increased over time. At Week 16, nearly half (46.0%) of patients achieved PASI ≤ 2. Two‐thirds (68.3%) of patients achieved PASI ≤ 2 and nearly half (47.6%) achieved PASI ≤ 1 at Week 52 (Figure 1B).

#### 3.2.2 Effect of Deucravacitinib Treatment on PASI Body Region and Plaque Characteristic Scores

**FIGURE 2:**
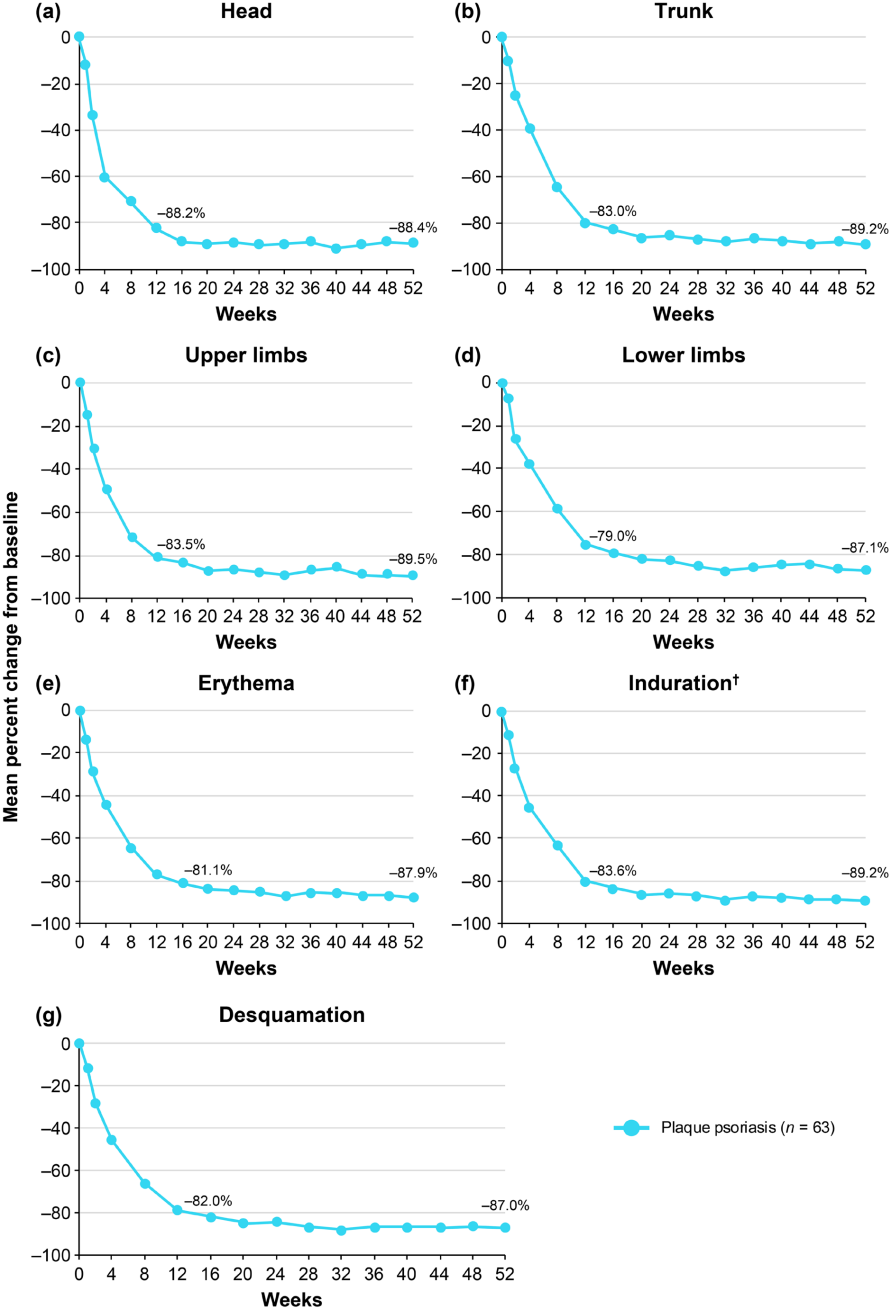
Mean percent change from baseline in (a–d) PASI body region and (e–g) plaque characteristic scores. Includes patients with a baseline value > 0. ^†^ *N* = 63 for all components, with the exception of induration, where *n* = 61. Modified baseline observation carried forward was used to impute missing data. PASI, Psoriasis Area and Severity Index.

Mean baseline body region and plaque characteristic scores used in PASI scoring are shown in Table 2. Deucravacitinib treatment was associated with substantial mean percent reductions from baseline across body regions (head, trunk, upper limbs, and lower limbs) and plaque characteristics (erythema, induration, and desquamation) used in PASI scoring; reductions, which were evident as early as Week 1, increased through Week 16 and were maintained or increased through Week 52 (Figure 2). Mean reductions from baseline ranged from approximately 80% at Week 16 to 90% at Week 52 across all body region and plaque characteristic scores. Substantial proportions of patients also achieved ≥ 75% reduction from baseline in each PASI body region and plaque characteristic score; reductions evident at Week 16 were generally maintained or increased through Week 52 (Table S1).

**TABLE 2:** Mean baseline body region and plaque characteristic scores used in PASI scoring.

	Plaque psoriasis (*n* = 63)
Body region score, mean (SD)	
Head/neck	22.5 (13.7)
Trunk	19.5 (11.0)
Upper limbs	17.7 (9.8)
Lower limbs	23.8 (12.4)
Plaque characteristic score, mean (SD)	
Erythema	7.7 (3.1)
Induration	6.7 (3.2)
Desquamation	6.7 (3.2)

Abbreviations: PASI, Psoriasis Area and Severity Index; SD, standard deviation.

#### 3.2.3 Effect of Prior Therapy for Psoriasis on Clinical Efficacy Response Rates in Deucravacitinib‐Treated Patients

**FIGURE 3:**
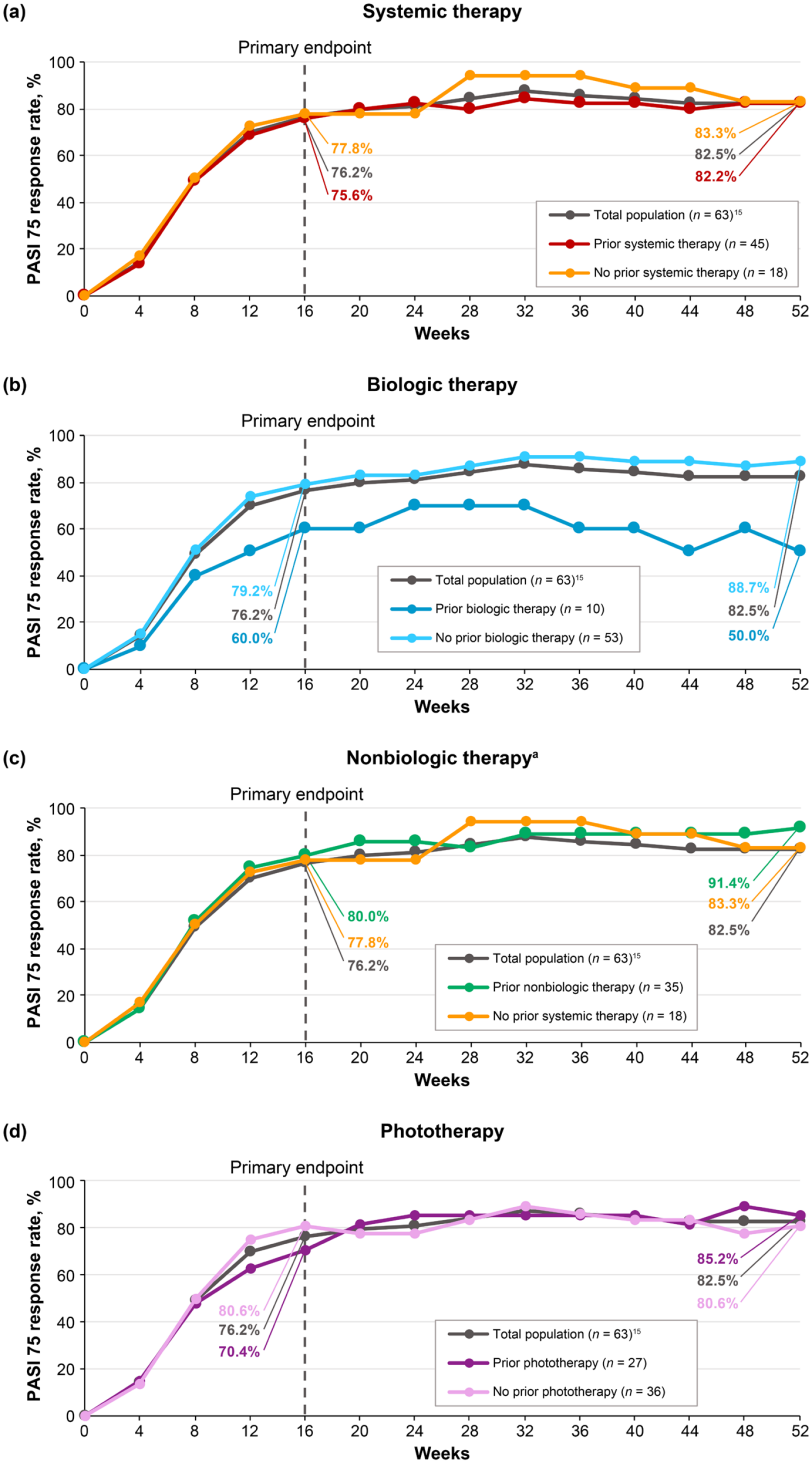
PASI 75 response rates by prior (a) systemic therapy, (b) biologic therapy, (c) nonbiologic therapy, and (d) phototherapy for psoriasis. ^a^Nonbiologic therapy includes the oral phosphodiesterase‐4 (PDE‐4) inhibitor apremilast; 26 of 63 (41.3%) patients had previously received apremilast therapy for plaque psoriasis. Nonresponder imputation was used to impute missing data. PASI 75, ≥ 75% reduction from baseline in Psoriasis Area and Severity Index.

**FIGURE 4:**
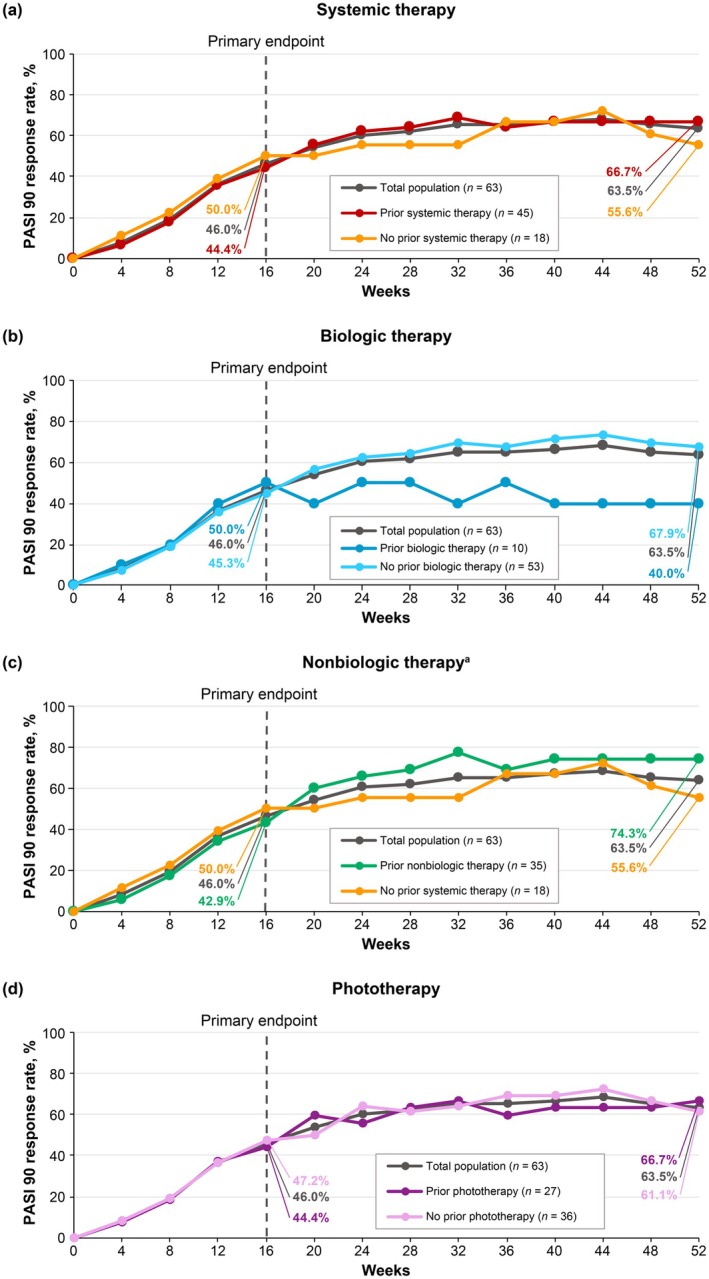
PASI 90 response rates by prior (a) systemic therapy, (b) biologic therapy, (c) nonbiologic therapy, and (d) phototherapy for psoriasis. ^a^Nonbiologic therapy includes the oral PDE‐4 inhibitor apremilast; 26 of 63 (41.3%) patients had previously received apremilast therapy for plaque psoriasis. Nonresponder imputation was used to impute missing data. PASI 90, ≥ 90% reduction from baseline in Psoriasis Area and Severity Index.

**FIGURE 5:**
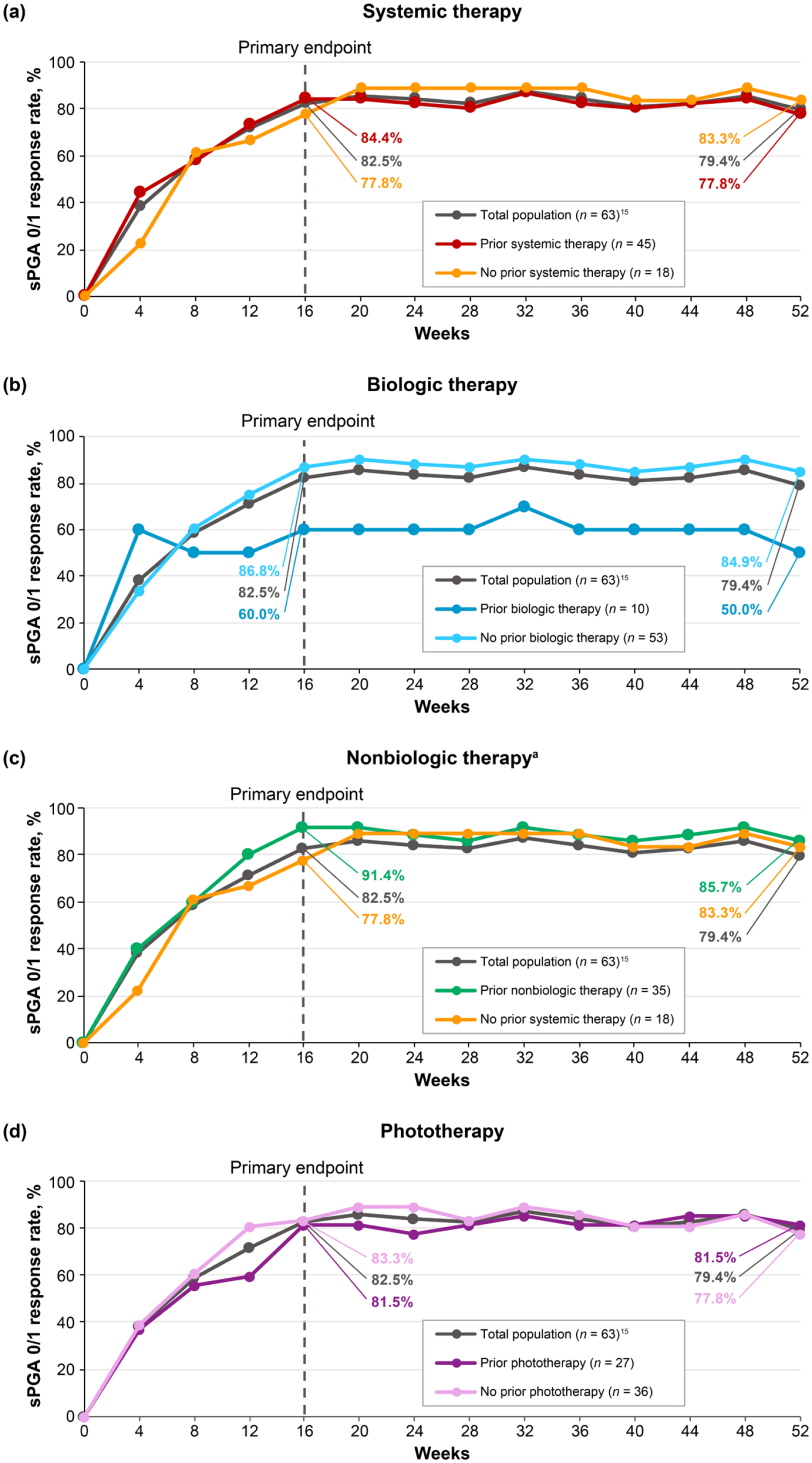
sPGA 0/1 response rates by prior (a) systemic therapy, (b) biologic therapy, (c) nonbiologic therapy, and (d) phototherapy for psoriasis. ^a^Nonbiologic therapy includes the oral PDE‐4 inhibitor apremilast; 26 of 63 (41.3%) patients had previously received apremilast therapy for plaque psoriasis. Nonresponder imputation was used to impute missing data. SPGA 0/1, static Physician Global Assessment score of 0 (clear) or 1 (almost clear) with a ≥ 2‐point improvement from baseline.

PASI 75 (Figure 3), PASI 90 (Figure 4), and sPGA 0/1 (Figure 5) response rates were generally similar in patients treated with deucravacitinib regardless of prior therapy (systemic [biologic and nonbiologic] therapy and phototherapy) for psoriasis. Based on the limited number of patients receiving biologic therapy (*n* = 10), it appears that response rates were slightly lower with prior biologic therapy compared with no prior biologic therapy.

#### 3.2.4 Efficacy of Deucravacitinib Treatment in Hard‐To‐Treat Areas

**FIGURE 6:**
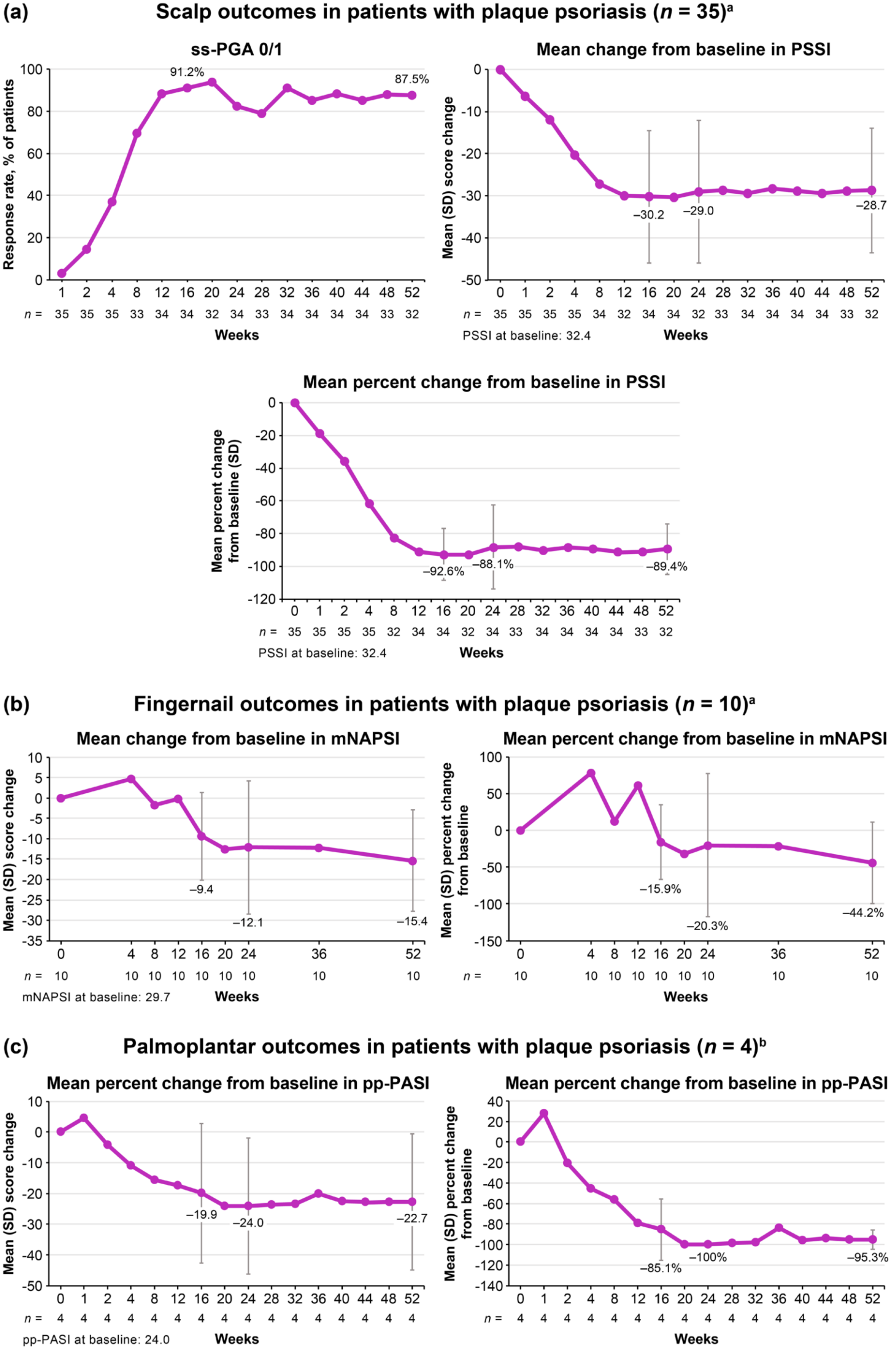
Outcomes in hard‐to‐treat types of psoriasis. (a) Scalp outcomes in patients with baseline ss‐PGA scores ≥ 3 (moderate to severe disease; *n* = 35). (b) Fingernail outcomes in patients with baseline PGA‐F scores ≥ 3 (moderate to severe disease; *n* = 10). (c) Palmoplantar outcomes in patients with baseline pp‐PGA scores ≥ 3 (moderate to severe disease; *n* = 4). Analyses were as observed. mNAPSI, modified Nail Psoriasis Severity Index; pp‐PASI, palmoplantar Psoriasis Area and Severity Index; PSSI, Psoriasis Scalp Severity Index; ss‐PGA 0/1, scalp‐specific Physician Global Assessment score of 0 or 1.

Among patients with plaque psoriasis and moderate to severe scalp involvement (ss‐PGA ≥ 3) at baseline, ss‐PGA 0/1 response rates increased from Week 1 through Week 16, and improvements were maintained through Week 52. Similarly, mean change from baseline and mean percent change from baseline in PSSI decreased from Week 1 through Week 16, and reductions were maintained through Week 52 (Figure 6A). Among the limited number of patients with moderate to severe fingernail psoriasis (PGA‐F ≥ 3) at baseline (*n* = 10), mean change from baseline and mean percent change from baseline in mNAPSI decreased from Week 1 through Week 16 followed by further reductions through Week 52 (Figure 6B). PGA‐F 0/1 response rates increased from Week 1 through Week 16, with further improvements evident through Week 52 (Figure S1A). Among the limited number of patients with moderate to severe palmoplantar psoriasis (pp‐PGA ≥ 3) at baseline (*n* = 4), mean change from baseline and mean percent change from baseline in pp‐PASI decreased through Week 16 and reductions were maintained through Week 52 (Figure 6C). pp‐PGA 0/1 response rates increased from Week 1 through Week 16, and improvements were maintained through Week 52 (Figure S1B).

### 3.3 Safety

Safety outcomes in patients with plaque psoriasis were previously described [15]. Total exposure over 52 weeks was 60.6 person‐years (PY). AEs were reported in 74.6% (exposure‐adjusted incidence rate [EAIR]/100 PY, 172.6) of patients; the most common AEs (occurring in ≥ 5% of patients) were nasopharyngitis (31.7%; EAIR/100 PY, 41.4), acne (7.9%; EAIR/100 PY, 8.8), periodontitis (6.3%; EAIR/100 PY, 6.8), and upper respiratory tract infection (6.3%; EAIR/100 PY, 6.8).

#### 3.3.1 Serious Adverse Events

Four patients (6.3%; EAIR/100 PY, 6.7) developed SAEs, reported as COVID‐19, pneumonia, normal pressure hydrocephalus, and asthma (Table 3). Most of these patients had risk factors for their specific SAE. All SAEs improved or resolved over time, and only the pneumonia event was considered related to treatment.

**TABLE 3:** Safety narratives for patients with SAEs or AEs resulting in treatment discontinuation.

AE	Patient narrative
SAE	
COVID‐19	A 44‐year‐old patient, with a medical history of Type 2 diabetes mellitus, dyslipidemia, hyperuricemia, and fatty liver disease, tested positive for COVID‐19 on Day 149 and was hospitalized on Day 150; the patient improved by Day 160; the event was not considered related to treatment by the investigator
Pneumonia	A 45‐year‐old patient was hospitalized with pneumonia on Day 363; this event resolved by Day 393 after the patient had rolled over into the POETYK long‐term extension trial; the event was considered related to treatment by the investigator
Normal pressure hydrocephalus	A 69‐year‐old patient, with a medical history of myocardial infarction, Type 2 diabetes mellitus, and lumbar disc herniation, was hospitalized due to normal pressure hydrocephalus on Day 263, which resolved by Day 277; the event was not considered related to treatment by the investigator
Asthma	A 50‐year‐old patient, with a medical history of asthma, hypertension, and cutaneous pruritus, visited an emergency department due to severe asthma on Day 92, which resolved by Day 113; the event was not considered related to treatment by the investigator
AE leading to discontinuation	
Psoriasis aggravation	A 23‐year‐old patient, with a medical history of dyslipidemia, had moderate psoriasis aggravation on Day 15 and discontinued treatment on Day 43; psoriasis aggravation resolved on Day 57; the event was not considered related to treatment by the investigator
Decreased neutrophil count	A 48‐year‐old patient, with a medical history of dyslipidemia, developed decreased neutrophil count on Day 141 and discontinued treatment on Day 278; the event was not considered related to treatment by the investigator

Abbreviations: AE, adverse event; SAE, serious adverse event.

#### 3.3.2 Adverse Events Resulting in Discontinuation of Deucravacitinib Treatment

Two patients (3.2%; EAIR/100 PY, 3.3) developed AEs (psoriasis aggravation and decreased neutrophil count) resulting in deucravacitinib treatment discontinuation (Table 3). Neither event was considered related to treatment.

#### 3.3.3 Shifts to Grade ≥ 3 Laboratory Abnormalities

As previously described, no clinically meaningful mean changes from baseline were observed in laboratory parameters over 52 weeks in patients with plaque psoriasis [15]. Most patients did not experience clinically meaningful shifts from baseline (CTCAE grade ≥ 3) in laboratory parameters through Week 52 (Table 4). CTCAE grade ≥ 3 triglyceride elevations occurred in three patients, neutrophil elevation occurred in one patient, and ALT and AST elevations each occurred in one patient. In general, patients had risk factors for these laboratory abnormalities, and some abnormalities were transient, with values returning to within normal limits, either spontaneously or after treatment interruption. The patient with the neutrophil reduction discontinued treatment, as described above.

**TABLE 4:** CTCAE grade ≥ 3 laboratory abnormalities, Weeks 0–52.

Laboratory parameter	Grade	Plaque psoriasis (*n* = 63)	
		Baseline, *n* (%)	Week 52, *n* (%)
Lymphocytes	3 4	0 0	0 0
Neutrophils	3 4	0 0	1 (1.6)^a^ 0
Platelets	3 4	0 0	0 0
Anemia	3 4	0 NA^b^	0^b^
Total cholesterol	3 4	0 0	0 0
Triglycerides increased	3 4	0 0	2 (3.2)^c^, ^d^ 1 (1.6)^e^
CPK increased^f^	3 4	0 0	0 0
Creatinine	3 4	0 0	0 0
ALT	3 4	0 0	1 (1.6)^g^ 0
AST	3 4	0 0	1 (1.6)^g^ 0

Abbreviations: AE, adverse event; ALT, alanine aminotransferase; AST, aspartate aminotransferase; CPK, creatine phosphokinase; CTCAE, Common Terminology Criteria for Adverse Events; SAE, serious adverse event.

^a^ The patient experienced a transient, asymptomatic, Grade 3 increase in neutrophils at Week 40, which the investigator considered treatment‐related and discontinued deucravacitinib treatment.

^b^ CTCAE version 5.0 has no designation of Grade 4 for hemoglobin per anemia criteria.

^c^ One event occurred at Week 16 (triglycerides, 7.5 mmol/L) in a patient with a history of dyslipidemia and Grade 1 hypertriglyceridemia at baseline (baseline triglycerides, 1.76 mmoL/L). Urinalysis showed high glucose levels (3+ mg/dL) at baseline, which remained elevated at the time of the event (Week 16, 3+ mg/dL) and at Week 52 (3+ mg/dL). This patient had ongoing Grade 2 hypertriglyceridemia at Week 52 (triglycerides, 3.53 mmol/L).

^d^ One event occurred at Week 52 (triglycerides, 6.25 mmol/L) in a patient with Grade 1 hypertriglyceridemia at baseline (triglycerides, 1.79 mmol/L). Urinalysis results in this patient were negative for glucose from baseline through Week 52.

^e^ This event occurred at Week 24 (triglycerides, 11.53 mmol/L) in a patient with a history of hyperlipidemia and Grade 1 hypertriglyceridemia at baseline (triglycerides, 1.84 mmoL/L). Urinalysis results in this patient were negative for glucose from baseline through Week 52.

^f^ CPK elevation was noted in one patient who had a Grade 2 elevation of ≥ 2.5× the upper limit of normal (ULN) at Week 40 (from grade 0 at baseline). No precipitating factor was identified, and the CPK elevation resolved without intervention after 29 days.

^g^ A patient had Grade 3 elevations in ALT and AST levels simultaneous with a diagnosis of a non‐SAE of infectious mononucleosis. The patient enrolled in the POETYK long‐term extension trial and by Week 4 of that trial, ALT and AST levels recovered to within normal limits.

## 4 Discussion

This in‐depth, post hoc analysis of POETYK PSO‐4 confirms that deucravacitinib is efficacious and well tolerated in Japanese patients with moderate to severe plaque psoriasis. Deucravacitinib improved absolute PASI beginning at Week 1, with improvements maintained through Week 52. Notably, deucravacitinib‐treated patients achieved clinically meaningful improvements in PASI thresholds, with nearly half (46.0%) achieving PASI ≤ 2, a real‐world treat‐to‐target threshold, by Week 16. Approximately two‐thirds had achieved this threshold by Week 24 (65.1%) and Week 52 (68.3%). Deucravacitinib improved psoriasis across all body regions (head, trunk, upper limbs, and lower limbs) and plaque characteristics (erythema, induration, and desquamation) used in PASI scoring. The proportions of deucravacitinib‐treated patients achieving ≥ 75% reduction from baseline were similar for each PASI body region and plaque characteristic score, indicating that deucravacitinib works equally well across body regions and kinds of plaques. Similar to absolute PASI, improvements in PASI component scores occurred as early as Week 1, increased through Week 16, and were maintained or increased through Week 52. Improvements ranged from approximately 80% at Week 16 to 90% at Week 52 across all PASI body region and plaque characteristic scores. Deucravacitinib was also efficacious through Week 52 regardless of prior use of systemic (biologic or nonbiologic) therapy or phototherapy for psoriasis. Based on a limited number of patients receiving biologic therapy (*n* = 10), response rates (PASI 75, PASI 90, and sPGA 0/1) were somewhat lower but still considered clinically meaningful in patients who had received prior biologic therapy compared with those who were naive to biologic therapy. This is consistent with analyses of systemic drug treatment patterns which have demonstrated that treatment persistence rates were lower among biologic‐experienced versus biologic‐naive patients, and that lack of efficacy was among the most common reasons for treatment discontinuation [28, 29, 30]. Based on a limited number of patients, deucravacitinib was efficacious through Week 52 in the hard‐to‐treat scalp and fingernail areas and also improved disease burden in the limited number of patients with moderate to severe palmoplantar involvement at baseline. Overall, these findings support the rapid onset and durability of response across multiple efficacy outcomes in Japanese patients with plaque psoriasis receiving deucravacitinib treatment.

The results of this post hoc analysis are supported by similar analyses of POETYK PSO‐1, which enrolled a limited number of Japanese patients; POETYK PSO‐2; and POETYK PSO‐3, which was an additional 52‐week, Phase 3 trial in Asian patients with moderate to severe plaque psoriasis [31, 32, 33, 34]. In POETYK PSO‐1 and PSO‐2, deucravacitinib achieved clinically meaningful reductions in absolute PASI and PASI thresholds that were statistically superior to placebo over 16 weeks (PASI ≤ 2: PSO‐1, 34.9% vs. 5.4%; PSO‐2, 30.1% vs. 4.3%, respectively; *p* < 0.0001 in both trials). Improvements in PASI with deucravacitinib treatment were evident as early as Week 1, increased through Week 16 and were maintained through Week 52 [33]. Deucravacitinib also achieved greater improvements than placebo in each body region and plaque characteristic used in PASI scoring [33]. The high efficacy rates achieved with deucravacitinib treatment across prior therapy subgroups in this POETYK PSO‐4 analysis are consistent with those previously reported in subgroup analyses of the pooled POETYK PSO‐1 and PSO‐2 population based on prior therapy for psoriasis [35]. Finally, deucravacitinib was more efficacious than placebo in improving scalp psoriasis in a pooled analysis of the POETYK PSO‐1 and PSO‐2 population, a Japanese subanalysis of the POETYK PSO‐1 population, and in the POETYK PSO‐3 population [31, 32, 34].

Similar to the overall analysis of POETYK PSO‐4, response rates were higher in this in‐depth analysis of the Japanese population compared with rates reported in similar analyses of the global population in POETYK PSO‐1 and PSO‐2 [15, 31, 33]. Higher efficacy in Japanese patients may be linked to lower body weight in this population [36, 37]. However, response rates in POETYK PSO‐3, a Phase 3 trial in Asian (predominantly Chinese) patients with higher body weight, were consistent with those reported in the subgroup analysis of Japanese patients in POETYK PSO‐1 [32, 34], suggesting that factors in addition to lower body weight may contribute to higher efficacy rates in the Japanese population.

Deucravacitinib treatment was well tolerated over 52 weeks in Japanese patients with moderate to severe plaque psoriasis. Incidence rates of SAEs and AEs resulting in treatment discontinuation were low. Shifts from baseline to grade ≥ 3 laboratory abnormalities at Week 52 were rare. These results are consistent with similar analyses of POETYK PSO‐1, PSO‐2, and PSO‐3, and the ongoing open‐label POETYK long‐term extension (NCT04036435) trial [33, 34, 38, 39].

Study limitations include the relatively limited number of patients receiving biologic therapy (*n* = 10), as well as the limited number with fingernail psoriasis (*n* = 10) and palmoplantar psoriasis (*n* = 4), making it challenging to reach definitive conclusions about the efficacy of deucravacitinib treatment in Japanese patients who have received prior systemic therapy or have hard‐to‐treat types of psoriasis. Additional limitations include the preponderance of male patients (76.2%) in this analysis, although this is consistent with the established prevalence of psoriasis in the Japanese population [40]. The POETYK long‐term extension trial will provide longer‐term, real‐world data about the efficacy and safety of deucravacitinib treatment in Japanese patients with moderate to severe plaque psoriasis.

In conclusion, the findings of this in‐depth analysis of deucravacitinib efficacy and safety in Japanese patients with plaque psoriasis provide valuable new information not included in the previously reported primary results of POETYK PSO‐4 [15]. Achievement of clinically relevant absolute PASI thresholds, effect of prior therapy, efficacy in hard‐to‐treat areas, and details about AEs of particular concern are presented to provide clinicians with a clearer picture of the degree and timing of onset of efficacy and potential AEs. This is a clinically relevant publication that will help identify appropriate Japanese patients for whom deucravacitinib treatment should be prescribed. The findings reported here provide additional support that deucravacitinib, a once‐daily oral TYK2 inhibitor, is an efficacious and well‐tolerated therapeutic option for Japanese patients with moderate to severe plaque psoriasis.

## Disclosure

Shinichi Imafuku and Yayoi Tada are Editorial Board members of *Journal of Dermatology* and co‐authors of this article. To minimize bias, they were excluded from all editorial decision‐making related to the acceptance of this article for publication.

## Ethics Statement

This study was conducted in accordance with Good Clinical Practice, as defined by the International Council for Harmonization and the Declaration of Helsinki. Institutional review board approval was received at each site, and all patients provided written informed consent. The study is included on the www.ClinicalTrials.gov website (NCT03924427).

## Conflicts of Interest

Yukari Okubo has received research funding from AbbVie, Eisai, Maruho, Shiseido, Sun Pharma, and Torii; honoraria as a speaker, consultant, and advisory board member from AbbVie, Amgen, Boehringer Ingelheim, Bristol Myers Squibb, Janssen, Kyowa Kirin Pharma, Leo Pharma, Lilly, Maruho, Novartis, Pfizer, Sanofi, Sun Pharma, Taiho, and UCB Japan; and honoraria as a speaker from Eisai, Jimro, Mitsubishi Tanabe, and Torii. Akimichi Morita has received honoraria as meeting chair/lecturer from AbbVie, AYUMI, Boehringer Ingelheim Japan, Celgene K.K., Eisai, Eli Lilly Japan K.K., Inforward, Janssen Pharmaceutical K.K., Kyowa Kirin, Maruho Co., Mitsubishi Tanabe Pharma, Nippon Kayaku, Novartis Pharma K.K., Taiho, Torii, and Ushio; has received clinical trial funding from AbbVie G.K., Eisai, Eli Lilly Japan K.K., Kyowa Hakko Kirin, Leo Pharma K.K., Maruho, Mitsubishi Tanabe Pharma, Novartis Pharma K.K., Taiho, and Torii; and has received consulting fees from AbbVie GK, Boehringer Ingelheim Japan, Bristol Myers Squibb, Celgene K.K., Eli Lilly Japan K.K., GlaxoSmithKline K.K., Janssen Pharmaceutical K.K., Kyowa Kirin, Maruho, Mitsubishi Tanabe Pharma, Nichi‐Iko, Nippon Kayaku, Novartis Pharma K.K., Pfizer Japan, Sun Pharma, Torii, and UCB Japan. Shinichi Imafuku has received grants and/or personal fees from AbbVie, Alexion, Amgen, Boehringer Ingelheim, Bristol Myers Squibb, Daiichi Sankyo, Eisai, Janssen, GSK, Kaken, Kyowa Kirin, Leo Pharma, Lilly, Maruho, Novartis, Sun Pharma, Taiho Yakuhin, Torii Yakuhin, and UCB. Yayoi Tada has received research grants from AbbVie, Amgen, Boehringer Ingelheim, Bristol Myers Squibb, Eisai, Jimro, Kyowa Kirin, Leo Pharma, Lilly, Maruho, Sun Pharma, Taiho, Tanabe‐Mitsubishi, Torii, and UCB; has received honoraria from AbbVie, Amgen, Boehringer Ingelheim, Bristol Myers Squibb, Eisai, Janssen, Jimro, Kyowa Kirin, Leo Pharma, Lilly, Maruho, Novartis, Pfizer, Sun Pharma, Taiho, Tanabe‐Mitsubishi, Torii, and UCB; and has received consulting fees from AbbVie, Boehringer Ingelheim, Bristol Myers Squibb, Janssen, Lilly, Maruho, Novartis, Taiho, and UCB. Katsuki Tsuritani and Katsuyoshi Habiro are employees of Bristol Myers Squibb K.K. Yanqiu Shao, Zoran Popmihajlov, Andrew Napoli, and Lauren Hippeli are employees of and shareholders in Bristol Myers Squibb. Mamitaro Ohtsuki has received honoraria and/or research grants from AbbVie, Amgen, Boehringer Ingelheim, Bristol Myers Squibb, Celgene, Eisai, Janssen, Kyowa Kirin, Leo Pharma, Lilly, Maruho, Mitsubishi Tanabe Pharma, Nichi‐Iko, Nippon Kayaku, Novartis, Pfizer, Sanofi, Sun Pharma, Taiho, Torii, and UCB.

## Supporting information


Data S1.

## Data Availability

The Bristol Myers Squibb policy on data sharing may be found at https://www.bms.com/researchers‐and‐partners/independent‐research/data‐sharing‐request‐process.html.

## References

[1] J. E. Greb , A. M. Goldminz , J. T. Elder , et al., “Psoriasis,” Nature Reviews. Disease Primers 2, no. 1 (2016): 16082, 10.1038/nrdp.2016.82. 27883001

[2] K. Kubota , Y. Kamijima , T. Sato , et al., “Epidemiology of Psoriasis and Palmoplantar Pustulosis: A Nationwide Study Using the Japanese National Claims Database,” BMJ Open 5 (2015): e006450. 10.1136/bmjopen-2014-006450 PMC4298108 25588781

[3] J. R. Burke , L. Cheng , K. M. Gillooly , et al., “Autoimmune Pathways in Mice and Humans Are Blocked by Pharmacological Stabilization of the TYK2 Pseudokinase Domain,” Science Translational Medicine 11 (2019): eaaw1736. 31341059 10.1126/scitranslmed.aaw1736

[4] P. Kulig , S. Musiol , S. N. Freiberger , et al., “IL‐12 Protects From Psoriasiform Skin Inflammation,” Nature Communications 7 (2016): 13466. 10.1038/ncomms13466 PMC5133729 27892456

[5] “Sotyktu [Package Insert],” (Bristol Myers Squibb, 2022).

[6] “Sotyktu [Package Insert],” (Bristol Myers Squibb K.K., 2022).

[7] “Sotyktu [Product Monograph],” (Bristol Myers Squibb Canada Co., 2022).

[8] “Sotyktu [Product Information],” (Bristol Myers Squibb Australia Pty. Ltd., 2022).

[9] “Sotyktu [European Summary of Product Characteristics],” (Bristol Myers Squibb EEIG, 2023).

[10] “Sotyktu [Package Insert],” (BMS Korea Pharmaceuticals Co., 2023).

[11] “Sotyktu [Package Insert],” (Bristol Myers Squibb (China) Investment Co. Ltd., 2023).

[12] S. T. Wrobleski , R. Moslin , S. Lin , et al., “Highly Selective Inhibition of Tyrosine Kinase 2 (TYK2) for the Treatment of Autoimmune Diseases: Discovery of the Allosteric Inhibitor BMS‐986165,” Journal of Medicinal Chemistry 62 (2019): 8973–8995. 31318208 10.1021/acs.jmedchem.9b00444

[13] A. W. Armstrong , M. Gooderham , R. B. Warren , et al., “Deucravacitinib Versus Placebo and Apremilast in Moderate to Severe Plaque Psoriasis: Efficacy and Safety Results From the 52‐Week, Randomized, Double‐Blinded, Placebo‐Controlled Phase 3 POETYK PSO‐1 Trial,” Journal of the American Academy of Dermatology 88 (2023): 29–39. 35820547 10.1016/j.jaad.2022.07.002

[14] B. Strober , D. Thaçi , H. Sofen , et al., “Deucravacitinib Versus Placebo and Apremilast in Moderate to Severe Plaque Psoriasis: Efficacy and Safety Results From the 52‐Week, Randomized, Double‐Blinded, Program for Evaluation of TYK2 Inhibitor Psoriasis Second Phase 3 Trial,” Journal of the American Academy of Dermatology 88 (2023): 40–51. 36115523 10.1016/j.jaad.2022.08.061

[15] S. Imafuku , Y. Okubo , Y. Tada , et al., “Deucravacitinib, an Oral, Selective, Allosteric Tyrosine Kinase 2 Inhibitor, in Japanese Patients With Moderate to Severe Plaque, Erythrodermic, or Generalized Pustular Psoriasis: Efficacy and Safety Results From an Open‐Label, Phase 3 Trial,” Journal of Dermatology 51 (2024): 365–379. 38268101 10.1111/1346-8138.17074 PMC11483964

[16] L. Puig , M. Dossenbach , L. Berggren , A. Ljungberg , and C. Zachariae , “Absolute and Relative Psoriasis Area and Severity Indices (PASI) for Comparison of the Efficacy of Ixekizumab to Etanercept and Placebo in Patients With Moderate‐To‐Severe Plaque Psoriasis: An Integrated Analysis of UNCOVER‐2 and UNCOVER‐3 Outcomes,” Acta Dermato‐Venereologica 99 (2019): 971–977. 31240322 10.2340/00015555-3245

[17] J. Yeung , M. Bourcier , M. J. Gooderham , et al., “Management of Moderate‐To‐Severe Plaque Psoriasis With Biologics: A Treat‐To‐Target Position Paper,” Dermatologic Therapy 35, no. 10 (2022): e15777, 10.1111/dth.15777. 35988045

[18] P. Gisondi , M. Talamonti , A. Chiricozzi , et al., “Treat‐To‐Target Approach for the Management of Patients With Moderate‐To‐Severe Plaque Psoriasis: Consensus Recommendations,” Dermatology and Therapy 11 (2021): 235–252. 33426634 10.1007/s13555-020-00475-8 PMC7859133

[19] F. Amatore , A. P. Villani , M. Tauber , M. Viguier , and B. Guillot , “French Guidelines on the Use of Systemic Treatments for Moderate‐To‐Severe Psoriasis in Adults,” Journal of the European Academy of Dermatology and Venereology 33 (2019): 464–483. 30793796 10.1111/jdv.15340 PMC6593704

[20] G. Carretero , L. Puig , J. M. Carrascosa , et al., “Redefining the Therapeutic Objective in Psoriatic Patients Candidates for Biological Therapy,” Journal of Dermatological Treatment 29 (2018): 334–346. 29099667 10.1080/09546634.2017.1395794

[21] H. Saeki , T. Mabuchi , A. Asahina , et al., “English Version of Japanese Guidance for Use of Biologics for Psoriasis (The 2022 Version),” Journal of Dermatology 50 (2023): e41–e68. 36582113 10.1111/1346-8138.16691

[22] S. K. Mahil , N. Wilson , N. Dand , et al., “Psoriasis Treat to Target: Defining Outcomes in Psoriasis Using Data From a Real‐World, Population‐Based Cohort Study (The British Association of Dermatologists Biologics and Immunomodulators Register, BADBIR),” British Journal of Dermatology 182 (2020): 1158–1166. 31286471 10.1111/bjd.18333 PMC7317460

[23] L. Grine , M. de la Brassinne , P. D. Ghislain , et al., “A Belgian Consensus on the Definition of a Treat‐To‐Target Outcome Set in Psoriasis Management,” Journal of the European Academy of Dermatology and Venereology 34 (2020): 676–684. 31749264 10.1111/jdv.16104 PMC7154521

[24] J. F. Merola , A. Qureshi , and M. E. Husni , “Underdiagnosed and Undertreated Psoriasis: Nuances of Treating Psoriasis Affecting the Scalp, Face, Intertriginous Areas, Genitals, Hands, Feet, and Nails,” Dermatologic Therapy 31 (2018): e12589. 29512290 10.1111/dth.12589 PMC6901032

[25] A. Oakley , “PASI Score,” https://dermnetnz.org/topics/pasi‐score# (2009).

[26] E. West , “What to Know About the PASI Score,” https://www.medicalnewstoday.com/articles/pasi‐score (2021).

[27] E. Mallard , “What Is the PASI Score for Psoriasis?” https://www.healthcentral.com/article/what‐is‐the‐pasi‐score (2025).

[28] Y. Tada , M. Komine , Y. Okubo , K. Habiro , K. Tsuritani , and A. Morita , “Treatment Patterns of Systemic Drug Use in Japanese Patients With Plaque Psoriasis: A Retrospective Chart Review,” Journal of Dermatology 51 (2024): 210–222. 38031882 10.1111/1346-8138.17038 PMC11484147

[29] T. Yanase , N. Tsuruta , K. Yamaguchi , et al., “Survival Rates of Systemic Interventions for Psoriasis in the Western Japan Psoriasis Registry: A Multicenter Retrospective Study,” Journal of Dermatology 50 (2023): 753–765. 36786158 10.1111/1346-8138.16737

[30] S. E. Thomas , L. Barenbrug , G. Hannink , M. M. B. Seyger , E. de Jong , and J. van den Reek , “Drug Survival of IL‐17 and IL‐23 Inhibitors for Psoriasis: A Systematic Review and Meta‐Analysis,” Drugs 84 (2024): 565–578. 38630365 10.1007/s40265-024-02028-1 PMC11190018

[31] A. Blauvelt , P. Rich , H. Sofen , et al., “Deucravacitinib, a Selective, Allosteric Tyrosine Kinase 2 Inhibitor, in Scalp Psoriasis: A Subset Analysis of Two Phase 3 Randomized Trials in Plaque Psoriasis,” Journal of the American Academy of Dermatology 90 (2024): 775–782. 38122848 10.1016/j.jaad.2023.11.060

[32] S. Imafuku , Y. Tada , L. Hippeli , S. Banerjee , A. Morita , and M. Ohtsuki , “Efficacy and Safety of the Selective TYK2 Inhibitor, Deucravacitinib, in Japanese Patients With Moderate to Severe Plaque Psoriasis: Subgroup Analysis of a Randomized, Double‐Blind, Placebo‐Controlled, Global Phase 3 Trial,” Journal of Dermatology 50 (2023): 588–595. 36882942 10.1111/1346-8138.16740

[33] M. Lebwohl , M. Gooderham , R. B. Warren , et al., “Deucravacitinib, an Oral, Selective, Allosteric Tyrosine Kinase 2 Inhibitor, in Moderate to Severe Plaque Psoriasis: Absolute PASI Outcomes Over 52 Weeks in the Phase 3 POETYK PSO‐1 Trial,” poster presented at Quadrennial World Congress of Dermatology, Singapore, July 3–8, 2023.

[34] J. Zhang , Y. Ding , P. Wang , et al., “Deucravacitinib, an Oral, Selective, Allosteric Tyrosine Kinase 2 Inhibitor, in Patients From China Mainland, Taiwan, and South Korea With Moderate to Severe Plaque Psoriasis: A Phase 3 Randomized Clinical Trial,” British Journal of Dermatology 192 (2025): 402–409. 39437312 10.1093/bjd/ljae406

[35] R. B. Warren , A. W. Armstrong , M. Gooderham , et al., “Deucravacitinib, an Oral, Selective Tyrosine Kinase 2 Inhibitor, in Moderate to Severe Plaque Psoriasis: 52‐Week Efficacy Results From the Phase 3 POETYK PSO‐1 and POETYK PSO‐2 Trials [Oral Presentation],” presented at The European Academy of Dermatology and Venereology Congress, September 29–October 2 (2021).

[36] A. Kisielnicka , A. Szczerkowska‐Dobosz , and R. J. Nowicki , “The Influence of Body Weight of Patients With Chronic Plaque Psoriasis on Biological Treatment Response,” Postepy Dermatologii I Alergologii 37 (2020): 168–173. 32489349 10.5114/ada.2020.94835 PMC7262805

[37] F. Pirro , G. Caldarola , A. Chiricozzi , et al., “Impact of Body Mass Index on the Efficacy of Biological Therapies in Patients With Psoriasis: A Real‐World Study,” Clinical Drug Investigation 41 (2021): 917–925. 34537921 10.1007/s40261-021-01080-z PMC8481196

[38] B. Strober , A. Blauvelt , R. Warren , et al., “Deucravacitinib in Moderate to Severe Plaque Psoriasis: Pooled Safety and Tolerability Over 52 Weeks From Two Phase 3 Trials (POETYK PSO‐1 and PSO‐2),” Journal of the European Academy of Dermatology and Venereology 38 (2024): 1543–1554. 38451052 10.1111/jdv.19925

[39] A. W. Armstrong , M. Lebwohl , R. B. Warren , et al., “Safety and Efficacy of Deucravacitinib in Moderate to Severe Plaque Psoriasis for Up to 3 Years: An Open‐Label Extension of Randomized Clinical Trials,” JAMA Dermatology 161, no. 1 (2025): 56–66, 10.1001/jamadermatol.2024.4688. 39602111 PMC11736510

[40] K. Kamiya , N. Oiso , A. Kawada , and M. Ohtsuki , “Epidemiological Survey of the Psoriasis Patients in the Japanese Society for Psoriasis Research From 2013 to 2018,” Journal of Dermatology 48 (2021): 864–875. 33580908 10.1111/1346-8138.15803 PMC8247979

