# Supplementary material for: Deucravacitinib, an Oral, Selective, Allosteric Tyrosine Kinase 2 Inhibitor, in Japanese Patients With Plaque Psoriasis: In‐Depth Analysis of Efficacy and Safety in the Phase 3 POETYK PSO‐4 Trial
Source: J Dermatol. 2025 Apr 30;52(6):953–66. doi: 10.1111/1346-8138.17744 (PMC12149362; doi:10.1111/1346-8138.17744)

**SUPPORTING INFORMATION**

**TABLE S1** Achievement of ≥75% improvement from baseline in PASI body region and plaque characteristic scores.

|  | **Plaque psoriasis**  **(*n* = 63)** | | |
| --- | --- | --- | --- |
|  | **Week 16** | **Week 24** | **Week 52** |
| Body region, % of patients achieving ≥75% improvement | | | |
| Head/neck | 85.2 | 86.9 | 77.0 |
| Trunk | 73.0 | 84.1 | 85.7 |
| Upper limbs | 76.2 | 81.0 | 81.0 |
| Lower limbs | 68.3 | 81.0 | 82.5 |
| Plaque characteristic, % of patients achieving ≥75% improvement | | | |
| Erythema | 76.2 | 79.4 | 84.1 |
| Induration | 79.4 | 82.5 | 81.0 |
| Desquamation | 71.4 | 82.5 | 82.5 |

Nonresponder imputation was used to impute missing data.
Abbreviation: PASI, Psoriasis Area and Severity Index.

**FIGURE S1** (**a**) Fingernail outcomes in patients with baseline PGA-F scores ≥3 (moderate to severe disease; *n* = 10). (**b**) Palmoplantar outcomes in patients with baseline pp-PGA scores ≥ 3 (moderate to severe disease; *n* = 4). Analyses were as observed. Abbreviations: PGA-F 0/1, Physician Global Assessment-Fingernails score of 0 or 1; pp-PGA 0/1, palmoplantar Physician Global Assessment score of 0 or 1.


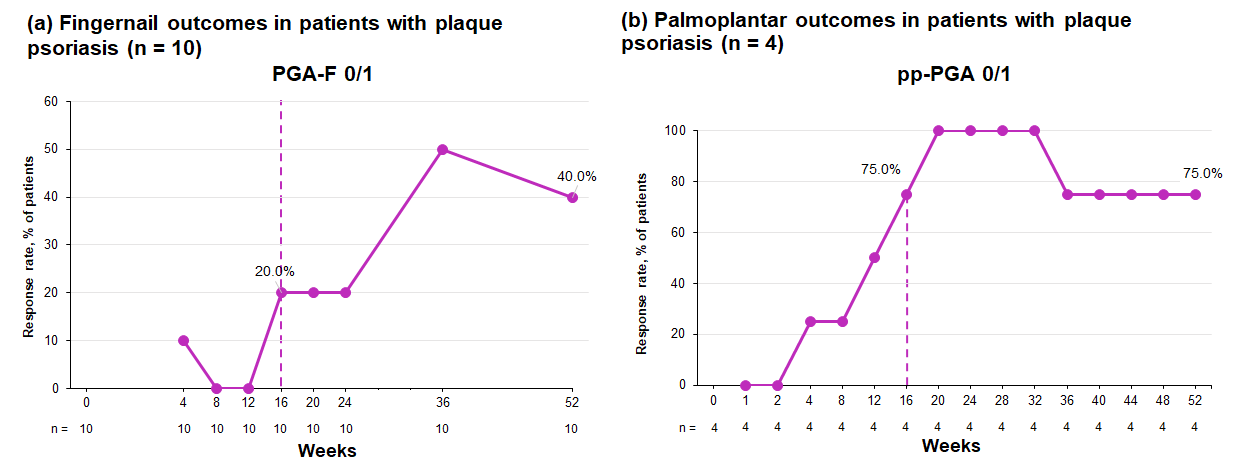

Supplement: Supplementary file 1 — Data S1. [file JDE-52-953-s001.docx]
